# Certain class of higher-dimensional simplicial complexes and universal *C*^∗^-algebras

**DOI:** 10.1186/2193-1801-3-258

**Published:** 2014-05-21

**Authors:** Saleh Omran

**Affiliations:** Faculty of Science, Taif University, Taif, Kingdom of Saudi Arabia; Faculty of science, Math. Department, KSA South valley university, Qena, Egypt

**Keywords:** Simplicial complexes, K- theory of *C*^∗^-algebras; Universal *C*^∗^-algebras

## Abstract

**Abstract:**

In this article we introduce a universal *C*^∗^-algebras associated to certain simplicial flag complexes. We denote it by it is a subalgebra of the noncommutative *n*-sphere which introduced by J.Cuntz. We present a technical lemma to determine the quotient of the skeleton filtration of a general universal *C*^∗^-algebra associated to a simplicial flag complex. We examine the *K*-theory of this algebra. Moreover we prove that any such algebra divided by the ideal *I*_2_ is commutative.

**2000 AMS:**

19 K 46

## Introduction

In this section, we give a survey of some basic definitions and properties of the universal *C*^∗^-algebra associated to a certain flag complex which we will use in the sequel. Such algebras in general was introduced first by Cuntz (2002) and studied by Omran (2005, 2013).

**Definition 1**. *A simplicial complex Σ consists of a set of vertices V*_*Σ*_*and a set of non-empty subsets of V*_*Σ*_, *the simplexes in Σ*, *such that:*

- *If s ∈ V*_*Σ*_, *then* {*s*} ∈ *Σ*.
- *If F ∈ Σ and ∅* ≠ *E* ⊂ *F then E* ∈ *Σ*.

*A simplicial complex Σ is called flag or full, if it is determined by its 1-simplexes in the sense that* {*s*_0_, …, *s*_*n*_} ∈ *Σ* ⇔ {*s*_*i*_, *s*_*j*_} ∈ *Σ for all* 0 ≤ *i* < *j* ≤ *n*.

*Σ* is called locally finite if every vertex of *Σ* is contained in only finitely many simplexes of *Σ*, and finite-dimensional (of dimension ≤*n*) if it contains no simplexes with more than *n*+1-vertices. For a simplicial complex *Σ* one can define the topological space |*Σ*| associated to this complex. It is called the “geometric realization” of the complex and can be defined as the space of maps *f*:*V*_*Σ*_ → [0, 1] such that  and *f*(*s*_0_) ..... *f*(*s*_*i*_) = 0 whenever {*s*_0_, …, *s*_*i*_} ∉ *Σ*. If *Σ* is locally finite, then |*Σ*| is locally compact.

Let *Σ* be a locally finite flag simplicial complex. Denote by *V*_*Σ*_ the set of its vertices. Define  as the universal *C*^∗^-algebra with positive generators *h*_*s*_, *s* ∈ *V*, satisfying the relations 

Here the sum is finite, because *Σ* is locally finite.

 is the abelian version of the universal *C*^∗^-algebra above, i.e. satisfying in addition *h*_*s*_*h*_*t*_ = *h*_*t*_*h*_*s*_ forall *s*, *t* ∈ *V*_*Σ*_. Denote by *I*_*k*_ the ideal in  generated by products containing at least *n* + 1 different generators. The filtration (*I*_*k*_) of  is called the skeleton filtration.

Let 

 be the standard *n*-simplex. Denote by  the associated universal *C*^∗^-algebra with generators *h*_*s*_, *s* ∈ {*s*_0_, …, *s*_*n*_}, such that *h*_*s*_ ≥ 0 and . Denote by  the ideal in  generated by products of generators containing all the . For each *k*, denote by *I*_*k*_ the ideal in  generated by all products of generators *h*_*s*_ containing at least *k* + 1 pairwise different generators. We also denote by  the image of *I*_*k*_ in . The algebra  and their *K*-Theory was studied in details in (Omran and Gouda 2012). For any vertex *t* in *Δ* there is a natural evaluation map  mapping the generators *h*_*t*_ to 1 and all the other generators to 0. The following propositions are due to Cuntz (2002).

**Proposition 1**. (*i*) *The evaluation map**defined above induces an isomorphism in K-theory*. (*ii*) *The surjective map**induces an isomorphism in K-theory, where**is the abelianization of*.

We can observe that *I*_*k*_ is the kernel of the evaluation map which define above so we can conclude that *I*_*k*_ is closed.

**Remark 1**. *Let Δ and**as above. Then*, *if the dimension n of* △ *is even and*, *if the dimension n of* △ *is odd*.

**Proposition 2**. *Let Σ be a locally finite simplicial complex. Then**is isomorphic to C*_0_(|*Σ*|), *the algebra of continuous functions vanishing at infinity on the geometric realization* |*Σ*| *of Σ*.

## Universal ***C***^***∗***^-algebras associated to certain complexes

Universal *C*^∗^-algebras is a *C*^∗^-algebras generated by generators and relations. Many *C*^∗^-algebras can be constructed in the form of universal *C*^∗^-algebras an important example for universal *C*^∗^-algebras is Cuntz algebras *O*_*n*_ the existence of this algebras and their *K*-theory was introduced by Cuntz (1981, 1984) more other examples of universal *C*^∗^-algebras can be found in (Cuntz 1993; Davidson 1996). In the following, we introduce a general technical lemma to compute the quotient of the skeleton filtration for a general algebra associated to simplicial complex.

For a subset *W* ⊂ *V*_*Σ*_, let *Γ* ⊂ *Σ* be the subcomplex generated by *W* and let  be the ideal in  generated by products containing all generators of .

**Lemma 1**. *Let**and**as above, then we have*

*Proof*. is generated by the images , *i* ∈ *V*_*Σ*_ of the generators in the quotient.

Given a subset *W* ⊂ *V*_*Σ*_ with |*W*| = *k* + 1, let 

Let  denote the ideal in  generated by products containing all generators , *i* ∈ *Γ*^′^, and let  denote its closure. If , then , because the product of any two elements in  and  contains products of more than *k* + 1-different generators, which are equal to zero in the algebra 

It is clear that  so that 

Conversely, let *x* ∈ *I*_*k*_ / *I*_*k*+1_. Then there is a sequence (*x*_*n*_) converging to *x*, such that each *x*_*n*_ is a sum of monomials *m*_*s*_ in  containing at least *k* + 1-different generators. Then  for some *W* and 

 The space  is closed, because it is a direct sum of closed ideals. It follows that 

Let now 

be the canonical evaluation map defined by 

 where  denotes the generator in  corresponding to the index *i* in *W*, in other words 

We prove that *π*_*W*_(*I*_*k*+1_) = 0. Since polynomials of the form 

 are dense in *I*_*k*+1_, it is enough to show that *π*_*W*_(*x*) = 0 for each such polynomial *x*. We have 

 since there is at least one *i*_*l*_ which is not in *W*. For this index . Thus *π*_*W*_(*x*) = 0. Therefore *π*_*W*_ descends to a homomorphism 

 Now we show that *π*_*W*_ is surjective as follows: Since *π*_*W*_(*I*_*k*+1_) = 0, we have Ker *π*_*W*_ ⊃ *I*_*k*+1_. It follows that the following diagram 

 commutes and  is well defined. This shows that  is a closed subalgebra in  and isomorphic to . We have . It is clear that Ker *π*_*W*_ is the ideal generated by *h*_*i*_ for *i* not in *W* and therefore  is generated by  for *i* not in *W*. This comes at once from the definitions of  and *π*_*W*_(*h*_*i*_) above and the fact that both are equal. We conclude that . This again implies that . Moreover the following diagram is commutative: 

 So,  is dense and closed in . Therefore  is injective and surjective.

As a consequence of the above lemma we have the following.

**Proposition 3**. *Let**and I*_*k*_*defined as above. Then we have an isomorphism*

*where the sum is taken over all k-simplexes △ in Σ*.

*Proof*. As in the proof of lemma 1 above with *Σ* = *Δ*, we find that: 

In the following we study the *C*^∗^-algebras  associated to simplicial flag complexes *Γ* of a specific simple type. These simplicial complexes is a subcomplex of the “non-commutative spheres” in the sense of Cuntz work (Cuntz 2002). We determine the *K*-theory of  and also the *K*-theory of its skeleton filtration. The *K*-theory of *C*^∗^-algebras is a powerful tool for classifying *C*^∗^-algebras up to their Projections and unitaries, more details about *K*-theory of *C*^∗^-algebras found in the references (Blackadar 1986; Murphy 1990; Rørdam et al. 2000; Wegge-Olsen 1993).

We denote by *Γ*^*n*^ the simplicial complex with *n* + 2 vertices, given in the form 

 and 

 Let 

 be the universal *C*^∗^- algebra associated to *Γ*^*n*^. The existence of such algebras is due to Cuntz (2002). It is clear that for any element , we have ∥*h*_*i*_∥ ≤ 1.

Denote by  the natural ideal in  generated by products of generators containing all *h*_*i*_, . Then we have the skeleton filtration 

 The aim of this section is to prove that the *K*-theory of the ideals in the algebras  is equal to zero. We have the following

**Lemma 2**. *Let**be as above. Then**is homotopy equivalent to*.

*Proof*. Let  be the natural homomorphism which sends 1 to . For a fixed  such that *i* ≠ 0^-^,0^+^, define the homomorphism 

 by *α*(*h*_*i*_) = 1 and *α*(*h*_*j*_) = 0 for any *j* ≠ *i*. Notice that . Now define , ,  The elements *φ*_*t*_(*h*_*j*_), , satisfy the same relations as the elements *h*_*j*_ in :

(i) *φ*_*t*_(*h*_*j*_) ≥ 0
(ii)
(iii)

We note that  and *φ*_0_ = *β* ∘ *α*.

This implies that 

 This means that  is homotopy equivalent to .

From the above lemma, we have , for ∗ = 0,1.

Now we describe the subquotients of the skeleton filtration in .

**Proposition 4**. *In the C*^∗^-*algebra**one has*

*where the sum is taken over all subcomplexes* △ *of* Γ^*n*^*which are isomorphic to the standard k*-*simplex* △ *and over all subcomplexes γ of* Γ^*n*^*which contain both vertices* 0^+^, 0^-^*and the second sum is taken over every subcomplex γ which contains both vertices* 0^+^,0^-^*and whose number of vertices is k* + 1.

*Proof*. We use Lemma 1 above. For every  with |*W*| = *k* + 1, we have two cases. Either {0^+^,0^-^} is not a subset of *W*, then *Γ* is a *k*- simplex, or {0^+^,0^-^} is a subset of *W*, then *Γ* is a subcomplex in *Γ*^*n*^ isomorphic to *γ*. This proves our proposition.

**Lemma 3**. *For the complex Γ*^*n*^*with n* + 2 *vertices*, *is commutative and isomorphic to*.

*Proof*. Let  denote the image of a generator *h*_*i*_ for . One has the following relations: 

For every  in  we have 

Hence  is generated by *n* + 2 different orthogonal projections and therefore .

**Lemma 4**.*I*_1_ / *I*_2_*in**is isomorphic to**in*.

*Proof*. From the proposition 4 above, one has 

 where △^1^ is 1-simplex, and 

 Since  is commutative because the generators of  commute (since ). We get 

**Lemma 5**. In , we have *K*_0_(*I*_1_ / *I*_2_) = 0 and 

*Proof*. By applying above lemma, and proposition 4, we have 

 The sum contain  1-simplex, △^1^ ≅ *C*_0_(0, 1). where *K*_0_(*C*_0_(0, 1)) = 0 and .

**Lemma 6**. *is a commutative C*^∗^-*algebra*.

*Proof*. Consider the extension 

and the analogous extension for the abelianized algebras.

The extensions above induce the following commutative diagram: 

 We have from 3 isomorphisms  and from 4 that , so 

**Lemma 7**. *C*^∗^-*algebra**is commutative and K*_∗_(*I*_2_) = 0, ∗ = 0, 1 *where I*_2_*is an ideal in**defined as in the above*.

*Proof*. is generated by three positive generators, . Consider the product of two generators, say . We have that  and  commute with , therefore also .

By a similar computation we can show that  and *h*_1_ commute. This implies that  is commutative. Therefore *I*_2_ = 0 in  Then, at once *K*_∗_(*I*_2_) = 0.
